# Oral health academics’ conceptualisation of health promotion and perceived barriers and opportunities in dental practice: a qualitative study

**DOI:** 10.1186/s12903-021-01508-0

**Published:** 2021-03-26

**Authors:** Stacey Bracksley-O’Grady, Karen Anderson, Mohd Masood

**Affiliations:** 1 La Trobe Rural Health School, College of Science, Health and Engineering, La Trobe University, Edwards Rd Bendigo, Victoria, 3550 Australia; 2 Rural Department of Community and Allied Health, La Trobe Rural Health School, College of Science, Health and Engineering, La Trobe University, Edwards Rd Bendigo, Victoria, 3550 Australia; 3 Department of Dentistry and Oral Health, La Trobe Rural Health School, College of Science, Health and Engineering, La Trobe University, Edwards Rd Bendigo, Victoria, 3550 Australia; 4 Dental Institute, University of Turku, Turku, Finland

**Keywords:** Health promotion, Oral health educators, Nominal group technique, Barriers to health promotion, Opportunities to health promotion

## Abstract

**Background:**

Oral diseases place a significant burden on individual and population health. These diseases are largely preventable; health promotion initiatives have been shown to decrease the disease rates. However, there is limited implementation of health promotion in dentistry, this could be due to a number of factors; the ethos and philosophy of dentistry is focused on a curative, individualised approach to oral diseases, confusion around health promotion as a concept. Oral health academics are well placed to implement health promotion, training of these professionals needs to include prevention, as training influences dental practice. However, there is a little understanding about how oral health academics (dental professionals who educate dental and oral health students) view health promotion. The aim of this exploratory study is to understand how oral health academics conceptualise health promotion and perceive the barriers and possible opportunities for health promotion implementation in dental practice.

**Methods:**

Nominal group technique (NGT), a highly structured face-to-face meeting, was conducted with 24 oral health academics to explore how they conceptualize health promotion and the barriers and opportunities for health promotion in practice. An additional 4 questions were emailed to oral health educators after the NGT meeting to gather additional data, 6 oral health academics were involved. The data was analyzed using thematic analysis.

**Results:**

Three board themes were identified: “Knowledge, ideas and concepts of health promotion”, “Challenges to health promotion”, “Opportunities for health promotion practice”. The oral health academics in this study discussed health promotion in a holistic way, however, health education and behaviour change were mentioned more than other aspects of health promotion. The structure of dental practice specifically the curative approach that underpins dentistry and the lack of funding, and value placed on health promotion could act as a challenge to health promotion being implemented in practice. There has been a shift towards prevention in dentistry, however the participants acknowledge there needs to be a change in the curative culture of the profession. Collaboration with other health professionals and using a common risk factor approach were the identified opportunities for health promotion practice.

**Conclusions:**

Oral health academics have a holistic understanding of health promotion, but still focus more on behavioural approaches which is common within dentistry. For a change to occur in health promotion practice a change in the structure, curative approach and funding model of dentistry is required. Collaboration with other health professionals is an opportunity to be capitalised on. Training of future dental professionals is the perfect place to start to implement the changes and opportunities for health promotion presented in this paper.

## Background

Oral diseases, including periodontal disease and dental caries, are a significant burden on a population’s overall health and wellbeing. There is well established evidence on the preventable nature of oral diseases. Although dental treatment has had very little effect on preventing oral diseases but much of the focus is still on treatment [1–4]. Health promotion initiatives, which focus on upstream approaches such as decreasing exposure of sugary foods, daily use of fluoridated toothpaste and the use of the systematic (water) and topical fluoride [5–9] have been shown to prevent oral disease and improve oral health. Contrary to the above evidence on effectiveness of health promotion initiatives in improving oral health, there is limited implementation of health promotion initiatives [1]. This situation has been attributed to the lack of understanding of health promotion within the dental field [2, 10, 11]. Additionally the ethos and philosophy of dentistry is focused on a downstream patient-centred, curative and rehabilitative approach to dental diseases [1–4, 12], leading to a focus on behavior change and health education [13].

This current downstream approach to dental care is unaffordable, ineffective and inefficient [4]. There is a growing call to action that the oral health workforce shifts from the individualistic, clinical downstream approach to a more upstream approach focusing on the underlying causes of dental diseases [14]. Although there are some very recent initiatives focusing on the upstream approaches for example International Centre for Oral Health Inequalities Research and Policy (ICOHIRP) [15] the majority of focus is still on downstream approaches [14].

For a shift to occur from curative downstream approach to preventive upstream approach, training of dental and oral health practitioners needs to be focused on health promotion and prevention. It is known that teaching oral disease prevention in oral health training has a significant influence on dental graduates positive attitudes towards prevention [16], and attitudes towards prevention influence practice once graduated [17]. Oral health academics are involved in the teaching of dental and oral health courses, are responsible for the delivery and training of dental graduates. Oral health academics play a key role in training graduates in developing preventive practice and a positive attitude towards oral health promotion and prevention. It has been evident in other health related fields that how health academics conceptualize and deliver health promotion to students can influence the practice of health promotion once graduated [18]. However, conceptualization of health promotion by oral health academics is not known and how this training can influence health promotion practice of dental professionals is limited. This exploratory study is phase one of a larger study which is exploring health promotion training within dental and oral health curriculum. The aim of this exploratory phase is to understand how oral health academics conceptualise health promotion and perceive the barriers and possible opportunities for health promotion implementation in dental practice.

## Methods

Ethical approval was gained from La Trobe research ethics committee (ethics number FHEC 14/234) prior to the commencement of the study.

### Study setting

The study was carried out at the 14th annual meeting of College of Oral Health Academics (COHA) in 2014 held at La Trobe University, Bendigo campus, Australia. The COHA is a collective of academics, researchers and clinical educators who teach the professions of oral health therapy, dental therapy and dental hygiene throughout Australia, Fiji, and Micronesia & New Zealand. The COHA holds annual meetings, which are used for professional development along with sharing ideas and resources on teaching oral health content.

### Recruitment of participants

An information pack about the study and invitation to participate in this study was sent to all the 56 expected attendees ahead of the COHA meeting via email. Out of 56 invited oral health academics 24 accepted to participate in the study.

### Data collection

There was time allocated in the COHA meeting for the face-to-face data collection session. Written consent was gained prior to data collection. Data was collected using a modified Nominal Group Technique (NGT). NGT was a method first used in the 1960′s in social psychological research and considered as a mixed methods approach with qualitative data generated from group discussions and quantitative data generated from the voting and ranking stage [19]. NGT is mainly used for item generation and allows for discussion to occur [20]. NGT usually included a highly structured face-to-face meeting where opinions from experts are captured and combined [21–23]. NGT was an appropriate methodology to address the aim of this study, to explore a broad range of opinions on how oral health academics conceptualize health promotion [20]. The benefits of NGT are twofold, participants have equal opportunity to present their views [24, 25] and the group process avoids problems associated with other group meetings, such as data being influenced by vocal members and participants conforming to group opinion [22, 24, 26]. This method was used due to the limited time we had with the participants at the conference. NGT eliminates problems with group dynamics that can be experienced with other group methods, it encourages participation of all group members and was useful to generate ideas in a structured process, enabling the researchers to gather a broad range of opinions in a limited timeframe.

NGT most commonly involves five stages [23]; introduction and explanation, silent generation of ideas, sharing ideas, group discussion and voting and ranking (Table 1) [23]. A modified NGT model with four-stage was used (introduction and explanation, silent generation of ideas, sharing ideas and group discussion) in this study. The researchers agreed there was no benefit to the research question for using the fifth stage of NGT (voting and ranking). As there is little known about how oral health academics conceptualize health promotion all ideas are important to present and hold equal weight.

**Table 1:** Stages of nominal group technique (adapted from Potter et al. [23])

Stages of NGT	Description of stage	How the stage was implemented in this study
1. Introduction and explanation	This stage provides an overview and purpose of the meeting to the participants and presents the questions that will be asked during the meeting	Participants were sent an email two days prior to the NGT taking place. The email contained an outline of the structure of the NGT, what online system was being used, what they needed to bring (a device either a laptop or phone to access Poll Everywhere) and the three questions that would be asked during the session
2. Silent generation of ideas	The questions are posed to the participants and they have time to write down all their ideas/responses. It is important in this stage that participants do not discuss or consult other participants with their ideas	The three questions were asked one by one and participants were given five minutes per question to submit their answers. Answers were captured via Poll Everywhere. Participants could submit their responses via a webpage or by text
3. Sharing ideas	Participants are asked to share their ideas they have written down in stage 2. All ideas are written down and there is no discussion or debate between the participants during this stage	For this study stage 2 and 3 occurred simultaneously. The responses were displayed instantly on an overhead projector so all participants could see the responses in real time. However, all participants were asked not to discuss what was displayed on the screen until the next stage. Instead the participants were encouraged to send further responses to Poll Everywhere
4. Group discussions	In this stage participants can discuss the ideas presented and seek further clarification. New ideas can be suggested but no ideas from the previous stages should be eliminated	Once all three questions were posed to the group and responses for the questions were collected, participants completed discussions in groups of four to five people. Participants were asked to discuss their responses and the responses from the group for each question. One person in the group was the scribe and they recorded key points of the discussions on an iPad
5. Voting and ranking	This stage involves prioritizing and ranking the ideas from the above stages. Discussions and group ranking can occur, or all participants can rank ideas separately	This stage was not included in this study

In the first stage of NGT (introduction and explanation stage), prior to the data collection session all participants were provided via email the instruction on the structure of the session and the questions that would be asked in the session. In the second NGT stage (silent generation of ideas stage), the participants were asked three semi-structured questions: What is health promotion? What health promotion could we do in practice that we are not already doing? What are the barriers for health promotion implementation in practice? These questions were developed based on the literature available on the knowledge of health promotion among health professionals [27, 28]. Participants’ responses were captured through Poll Everywhere, (see Table 2 for responses generated) [29]. Poll Everywhere is an online an audience response system, which allows questions via polls to be displayed to the audience. All polls are assigned a unique code, which participants use to respond to the polls via a webpage or a text message. In the third NGT stage (sharing ideas stage), the responses from the polls were then displayed on a PowerPoint for the participants to see. Poll Everywhere was used again in this stage, it allowed the data to be presented to the rest of the group instantly and enabled the other participants to see all responses. In the fourth stage (group discussion stage), participants were divided into self-selected groups of four to five people to discuss the questions and responses collected in the previous stages. The ideas that were generated from this discussion were noted via a scribe (one participant from each group) on an iPad. These discussions were not audio recorded as the space where the data collection occurred had all groups in one space and clear audio recording could not be possible.

**Table 2:** Responses of the participants captured through Poll Everywhere

Poll question	Responses (presented as they were sent into Polleverywhere)
What is health promotion?	Is multi-layered Going beyond individual health education and looking at the influencing factors that contribute to personal health choices changing the conditions that influence health and allow people to control their lives—culture, environments, supports, policies Changing individual choices on behaviour related to health Promoting healthy choices by creating an upstream approach. Creating healthy public policy to create supportive environments Provide individuals and communities with information and the tools to improve health literacy, so they can make choices/ changes to improve over all health. Social determinants of health and the social contexts need to be considered in the development and implementation of any health promotion activities The process of enabling people to take control over their own health Combination of educational, political and environmental factors contributing to individual and community health. Health promotion aids to empower individuals and communities to take control of their own health. It's a multidisciplinary approach which entails social determinants of health, the common risk factor approach to health and health advocacy Giving information to an individual or group that is relevant to improving their well being Providing oral health messages to enable individuals and public to make informed choice about their health Raising awareness of health and well being sharing good health messages providing information and strategies to enable healthy lifestyle changes to individuals and communities from best evidence based research and practice Educating individuals, groups and the wider community on living well, improving health and making better lifestyle choices Preventing disease at a community, not individual level. Empowering people to ensure health choices are positive engaging with the community to deliver messages that may improve health outcomes providing information to empower people to make healthy choices is the action of improving individual and community health by applying measured approaches Educating people about healthy alternatives, so they are motivated to make an informed choice about their health A group of strategies that improve health and well being of the individual or community group Sharing health messages with communities and groups increasing knowledge and empowering communities to change health behaviour Developing and delivering health messages Delivering health messages to the community
What health promotion could we do in practice that we are not already doing?	Decent effective tailored behavioural interventions, collaborating with other health organisations to incorporate oral health, advocacy- talking up oral health To further develop interprofessional sustainable health promotion project work Focus more on social determinants of health and community outreach COHA2 actively working with health professionals, integrating oral health as an underpinning thread of all health promotion … Getting back to 'we More community awareness of healthy options. Making healthy choices more attractive. Enabling at risk groups within the communities. Interdisciplinary cooperation regarding wholistic health promotion Working more heavily in marginalized communities, taking students out of the formal clinical environment Try to better educate GP's actively working with health professionals to integrate oral health as an underpinning thread of all health promotion … Getting back to 'we have Capacity building of non-dental and non-health (e.g. Educators) professionals to deliver oral health messages Continuing support from Local Health Districts or communities when there is lack of cohesion In practice it is at times difficult for management to see value in a operator taking time out to provide health promotion to the community Integrating health promotion with other faculties within the university large scale media promotion—television/radio etc.—single, targeted, collective message work with other allied health professionals Universities should become health promoting environments e.g. Healthy together Victoria Achievement program Make a video aiming it at secondary school students and ask schools to integrate it into their health promotion plans Routine ethics approval for students projects to enable the students' research to be placed in the academic arena Work with other groups, health and community, and deliver messages along side pre-organised events more collaboration with other health disciplines to create an wholistic approach Integrating oral health messages within existing primary/secondary/tertiary School curriculum Linking health promotion strategies between BOH students and MOD students At university we should encourage inter professional practice, mix student cohorts, integrate health students use social media in private practice to foster community health for patients Engaging with health services outside dental and oral health Using social media as a platform for health promotion Risk assessment for communities rather than individuals Focused individual and community approaches based on accurate risk assessment
What are the barriers for health promotion implementation in practice?	Outcome measures not always tangible Govt needs to quantify distribution of public funding Does not have high importance in practice Health promotion does not produce instant measurable results. Therefore unable to measure benefit Mutually beneficial student placements Public fear of being told off Public not interested Lack of understanding people's needs in order to deliver effective and appropriate oral health promotion Clinical efficiency valued and rewarded as able to be measured Challenges engaging communities in health promotion activities The dominance of the bio medical model of health care Insurance rebates for health promotion interventions Limited public resources prioritised on treating current disease first public perceptions of the value of preventive/health promoting interventions Limited time and importance placed on health promotion An inability to value the relationship building elements of good health promotion Expectation that the OHTherapist role is in the mouth. No time allocation, no monetary rewards Not seen by the dental profession as been 'core business' In private practice, time spent needs to equal revenue Private practice employers want "bums on seats" not community service Time involved in planning and delivering health promotion activities Lack of research demonstrating cost effectiveness Lack of continued funding for projects Resources AND an overload of 'health messages' generally the population become complacent Lack of opportunities and support for clinicians to participate in health promotion activities Health promotion is deemed as less prestigious than clinical practice Limited time and money Lack of continuity of care due to new organisational structures Token gestures in practice due to lack of overall HP strategy. Need a policy making role in health administration Pressure from employers to perform at the expense of HP.—$$$ on the table Some students don't think it is important, focussing too on perfecting clinical surgical treatment Time and cost Lack of remuneration, time, confidence High patient workloads Cultural barriers Private practice—cost & time Funding systems Cost Knowledge gap by managers in private and public sectors. HP is not audited, poorly renumerated, poorly included in CPD course

### Data analysis

The data collected using Poll Everywhere was downloaded into excel spreadsheets from the Poll Everywhere website. The key points noted down by scribes on iPad’s during group discussion phase were emailed to the research team. All files were imported into Nvivo 12 and named after the question number and source type (Poll Everywhere or group discussion). The data was read and re-read so the researchers started to familiarize themselves with the emerging themes. Initial codes were generated. We developed a code manual with definitions for all codes. Individual extracts of text were coded into as many different themes as deemed relevant. Allocation of these codes into potential broad themes started. However, after this initial thematic analysis and a meeting between the research team, it was agreed that further clarification and depth of some of the key findings was needed. The researchers sent four additional questions along with the initial findings from stage one, via email, to participants. The questions were; Overall health promotion was defined and viewed holistically; however, health education and behaviour change were mentioned more times than other strategies. Do you feel this is true representation of how health promotion is seen within dentistry? Collaboration was a strong theme that came out of analysis when talking about opportunities for health promotion. How can our profession capitalise on this opportunity? Are there any barriers which you feel are relevant that have not been mentioned? There were quite a few barriers mentioned, what are some strategies that could overcome some of the barriers? The email was sent to 24 participants, six participants responded.

Data collected from the email responses were imported into Nvivo 12 and named after the question number and source type (email) to be consistent with the first data set. The research team then went through both data sets and began initial coding again using existing codes and developing new codes. Then a process began of reviewing and revising the codes to organise data into themes. The broad (parent) themes were deductively coded from the initial research questions. Diagramming was used to visually represent the themes and sub themes and to explore the relationships between the themes. Miscellaneous codes that did not fit within the themes or subthemes were kept in free nodes. Four themes and sub-themes were identified.

Further refinement and development of the themes was undertaken by the research team which resulted in four themes being condensed into three.

## Results

Twenty-four oral health academics participated in the nominal group and six participants responded to questions sent via email. The participants were all involved in the teaching of oral health students, roles varied from clinical educators, lecturers, subject and course coordinators. Due to the limited time allocated to the data collection session within the conference and to make sure the research team had sufficient time to conduct the NGT, specific demographics of participants were not collected. Thematic analysis of the participant responses demonstrates positive views about health promotion and three main themes were identified: “Knowledge, ideas and concepts of health promotion”, “Challenges to health promotion”, “Opportunities for health promotion practice”. A brief description of the three themes can be found in Table 3. These themes are reported below using participant words (in italics) to illuminate the themes. The data will be identified with a tag (Poll Everywhere, group discussion or email), which indicates where the data was collected.

**Table 3:** Description of themes identifies in thematic analysis of participants responses

Theme	Sub-themes	Theme summary
Knowledge, ideas and concepts of health promotion		Health education is seen as the main part of health promotion within dentistry This reliance on behavioural approaches and information giving could be attributed to dental professionals feeling more competent in health education rather than other health promotion strategies There has been a shift towards prevention in the dental profession however, this is still some work to be done in the space Health promotion strategies that could be implemented in practice include utilising social media platforms
Challenges to health promotion	Structural Resources Personal Cultural	Current structure of dental practice is focussed more on the biomedical model rather than preventative model of health Dental and other health professionals work in silos which creates a barrier for collaboration Clinical work is the main priority due to the funding model of dental care There is a lack of funding towards health promotion by governments and private health insurance This lack of funding means there is limited time for dental professionals to spend on health promotion There is a low value placed on the importance of health promotion by both patients and managers of dental practice There is a prestige around restorative dental work, but the same cannot be said about health promotion A change in culture away from the biomedical/curative approach is needed This change needs to happen at a range of levels for how dental practice is structured to the education of dental professionals at university
Opportunities for health promotion practice	Collaboration Common risk factor approach Capacity building	Risk factors of oral disease are shared with other health conditions so there is an opportunity to join forces with other health professionals to address these Multidisciplinary practice is an opportunity that needs to be capitalised on in the dental field. This needs to occur both within the clinical environment and outside of the clinic Dental professionals need to also collaborate with key members of the community

### Theme 1: knowledge, ideas and concepts of health promotion

All of the participants were able to identify a range of different approaches and ways to undertake health promotion, which included advocacy, working with communities, behaviour change, empowering, looking at the social determinants of health and education. However, collectively education, behaviour change and raising awareness of oral health issues were mentioned more times than the other health promotion strategies.

“Providing oral health messages to enable individuals and public to make informed choice about their health”—Poll Everywhere.

Participants were asked to comment on whether they believed that this was a true representation of health promotion within dentistry when further explanation was sort through email and all the participants agreed.

“Dentistry does see education & behaviour change as the main idea of health promotion”- question sent via email.

A reason to explain this reliance on behavioural approaches is that clinicians feel more competent and confident in behaviour change and feel this is where they will make the biggest impact. Another factor that can influence this is how dental practice is structured, health education and behaviour change approaches work well in clinical practice where dental professionals have time one on one with patients.

“This is the area where clinicians feel they can add most benefit to behaviour change and that they are competent in this aspect of oral health promotion.” question sent via email.

“We spend far more time chair side than actually trying to make policy change” question sent via email.

All participants discussed the progress made towards prevention in dentistry but did acknowledge that there was still more work that was needed. Some participants recognised that this change would take time and would most likely be a generational change.

The participants discussed possible opportunities for health promotion in practice. These opportunities included; collaborating with health professionals and primary and secondary educators, capacity building for professionals outside of dentistry and innovative strategies including the use of social media and mass media.

“Using social media as a platform for health promotion”- Poll Everywhere.

### Theme 2: challenges to health promotion

Participants discussed how dental practice is organised and structured as an influence on health promotion within the field. Currently a curative based treatment approach underpins the field of dentistry and oral health. This approach means more emphasis is placed on treatment than prevention.

“Private practice employers want "bums on seats" not community service”-Poll Everywhere.

“Biomedical approach supported by agenda of professional guilds” – group discussions.

Another structural challenge raised by participants is working in silos, which especially impacted on collaborating with other health professionals. Participants raised the issue of the lack of opportunities when working clinically due to health professionals tending to work in silos.

“Each health profession sees their area as more important (work in silos)”—question sent via email.

There is a lack of funding (government and private insurance) for health promotion within dentistry. Several participants mentioned the structure of funding within dentistry, which limits health promotion initiatives and promotes clinical treatment. Government funding prioritises treating disease rather than preventing disease as health promotion does not produce instant measurable results.

“[No] insurance rebates for health promotion interventions” – Poll Everywhere.

“Limited public resources—prioritised on treating current disease first” – Poll Everywhere.

“Health promotion does not produce instant measurable results. Therefore, unable to measure benefit- Poll Everywhere.

Due to the lack of funding participants felt there is limited time given to health promotion in practice and they required more time than they are given to plan and implement health promotion. As there is little to no funding provided by private health insurance for health promotion in clinical dental care, limited time can be spent on it. Time spent in clinical practice needs to equal revenue raised.

“In practice it is at times difficult for management to see value in a operator [sic] taking time out to provide health promotion to the community”- Poll Everywhere.

Another factor mentioned by participants was the lack of value placed on health promotion within dentistry. Participants spoke about the public not being interested in health promotion and that they did not see the value for money in health promotion compared to treatment. A discussion also centred around the lack of prestige of health promotion interventions. Participants felt that clinical practice is view as prestigious where there is not the same esteem placed on health promotion.

“Lack of value placed on health- at the patient level, at a managerial level- public and private practice”- group discussion.

“Health promotion is deemed as less prestigious than clinical practice” – Poll Everywhere.

Participants discussed the need for the profession to move away from the biomedical approach towards a preventative/population approach. This move would need to occur within dental practice and also in university training.

“The Dental profession as a whole still needs to acknowledge the necessity to reorientate the health care system to a preventive approach rather than a curative approach”—question sent via email.

### Theme 3: opportunities for health promotion practice

Collaboration was identified by the participants as an important factor of health promotion and saw it as an opportunity to be capitalised on within dentistry. Participants highlighted the need for dental professionals to work with other professionals (allied health and education) in order to provide a more holistic approach. Most participants acknowledged that the risk factors for oral disease are shared with other health conditions, therefore collaborating with other health professionals to address the risk factors rather than conditions themselves would be beneficial for everyone. It was stressed by participants to make multidisciplinary practice work they need to build trust and collaboration needs to occur not just on dental issues but other health issues.

“Actively working with health professionals, integrating oral health as an underpinning thread of all health promotion … Getting back to 'we’”—Poll Everywhere.

“Interdisciplinary relationships will help deliver health promotion that has a common risk factor approach”—question sent via email.

Although participants were interested in collaborations with practitioners outside of oral health, they were also interested in collaborating more with other oral health professionals and community members. Participants acknowledged the need to develop relationships with influential members of the community so that health promotion efforts would be embraced by the community.

“Linking health promotion strategies between BOH [Bachelor of Oral Health] students and MOD [Dentistry] students”—Poll Everywhere.

## Discussion

This exploratory study investigated how health promotion is conceptualised by oral health academics and the possible challenges and opportunities to the implementation of health promotion within clinical practice. This study is phase one of a larger study and the results provide the researchers with an understanding of what oral heath academics think about health promotion, which will inform the other phases of the study. The results help to provide an understanding of where health promotion curriculum is coming from in dental education and will inform the next phase which is a mapping exercise of oral health and dental health promotion curriculum in Australia. The curriculum mapping and this phase will then be used to inform the final phase which will explore these initial concepts in more detail with academic staff and graduates of dental and oral health programs.

Participants identified health promotion, as a broad range of activities at an individual, community and population level. The participants within the study were able to state and identify the key ideas that are associated with health promotion and their understanding of health promotion reflected the breadth of health promotion strategies recognised in key health promotion planning frameworks such as the Ottawa Charter and the health promotion continuum [30]. The understanding of the term health promotion has varied widely within dentistry, so it is positive that the participants of this study demonstrated an understanding of health promotion [31]. However, participants in this study were primarily a part of the education of dental therapists, dental hygienists and oral health therapists, not dentists. Dental therapists and hygienists are known in the dental field as preventative professionals with public health and behavioural sciences at the core of the profession [32]. If dentists and educators who primarily teach into dental degrees were included this may have changed how health promotion was viewed/defined.

Although participants acknowledged the wide-ranging nature of health promotion, the most mentioned theme or strategy in this definition was health education and behaviour change. This is not surprising as it is well documented that dentistry has a heavy reliance on behaviour change and health education for prevention [2, 14, 31, 33]. This behavioural approach is not incorrect, but is limited in effectiveness, unless combined with upstream approaches [2, 34].

A reason cited for the reliance on behaviour change/ health education is that dental professionals feel more confident and competent in this area of health promotion.

Sunnell and colleagues [35] study supports this finding, citing the dental hygiene graduates felt more confident in health promotion at an individual level rather than a community level. Dental professionals may feel more confident in behavioural approaches to health promotion, as there has been evidence to support the increased amount of time spent on a topic in university education increases professional confidence [35]. Studies, which have reported on the health promotion preparation of oral health professionals, have highlighted that the training focuses mainly on education or behaviour change approach, such as, providing oral hygiene instructions to patients in hospitals and delivering health education sessions [36–38]. This is not unsurprising as the culture of dentistry is focused on individuals and behaviour change [2]. Another influence on the of health promotion training is health promotion competencies set out by the accrediting bodies of dental educational institutions. Competencies are statements which set out the basic level of knowledge, skills, behaviours necessary for a graduating dental professional and act as a benchmark for universities curriculum [39]. These competencies relate to all aspects of professional activity such as clinical skills, communication, professionalism and prevention/ health promotion [39, 40]. In Australia there has been a revision of these competencies, with a reduction in the level of knowledge graduates need in health promotion [40]. However, for in Europe there has been an expansion of health promotion competencies for European dentists [39, 41].

Participants within this study held positive views towards prevention and the shift that has started to occur in dentistry, however they acknowledged that further work needs to occur. For change to occur, the curative culture of dentistry needs to be challenged and needs to happen on all levels [42]. The Lancet published a whole series on “oral health matters” with two major papers highlighting the key issues facing dentistry [42]. One of the issues was the need for a radical restructure of oral health systems towards an upstream preventative focus which is responsive to the populations needs [14]. Another area that can influence this cultural shift is within higher education. By including health promotion in the curriculum of dental and oral health programs students can start to see the relevance of this, the role they play and develop the attitudes, knowledge and skills needed to implement the breadth of health promotion intervention to improve oral health. Which will be explored in the future phases of this research study.

Participants highlighted, the ethos of dentistry which focuses on a curative approach to dental diseases, as an influence on health promotion practice within the clinical environment. This philosophy is one of an individual, behavioural, curative approach, which is reactive rather than proactive [2, 12, 14]. It is well documented that this approach is the default in dentistry [1, 3, 4, 12] and was supported by the participants in this study. Sbaraini [1] found that dentists define their professional identity as performing surgery, therefore they felt their job was to intervene and to mechanically repair and restore teeth. The ethos of dentistry does not fit well with the philosophy underpinning health promotion. Health promotion philosophy is focused on health as a positive concept and focuses on improving health via a range of strategies that goes beyond the health care system [43]. Richards [44] reported dentists value the restorative paradigm over the preventative one, while, participants in this study, acknowledged the restorative paradigm was the default in practice, held positive views towards both approaches. This curative approach is reinforced in dental practice as the funding model prioritises clinical treatment over prevention.

There is a lack of financial support for health promotion with dentistry. The dental field in most developed nations is structured on the fee-for-service basis meaning restorative treatment attracts a larger sum of money than providing toothbrushing instructions. Consequently, undertaking health promotion is discouraged within this system [45]. This barrier occurs in most developed nations, as the funding model for dental care is similar between countries [46]. Participants highlighted the lack of funding from private insurance providers to be a barrier to implementation of health promotion in practice. Participants also mentioned the way clinical practice is structured means time spent needs to equal revenue. The tension between being profitable and providing ethical dental care (which includes prevention) is supported by other studies [44, 47].

Little value is placed on health promotion within dental practice. Participants within this study expressed this view and previous studies support this finding [1, 2]. Dyer and Robinson [45] reported participants viewed health promotion and preventative practices to be unrewarding and therefore were not valued. This lack of value acts as a barrier to health promotion implementation [48]. A reason that could be attributed to this lack of value is the low or no monetary figure placed on health promotion interventions [17] or the lack of prestige around preventative work. Participants highlighted that clinical work is deemed more prestigious than preventative work. This could be attributed to greater value placed on the clinical paradigm and dentists seeing a move towards prevention as a devaluing of their restorative skills [44].

For health promotion, beyond health education. to be embedded as part of dental practice, there needs to be a shift from focusing on the barriers to health promotion, to how these can be overcome. Collaboration was one of the major ideas cited by participants that could be capitalised on. Participants in this study discussed addressing the risk factors that are shared between oral diseases and other conditions as a way of working with other health professionals. This common risk factor approach is not a new idea within dentistry [49–51] and is encouraged and supported by the World Health Organization [52]. However, dentistry has struggled with isolation from other professions [53], and it has been mentioned as a barrier when trying to promote oral health [46, 54]. There is a well-documented historical divide between dentistry and other health professions which has been reinforced through legislation, education and service delivery [55]. Oral health professionals tend to work in silos, separate from other health professionals, which was mentioned by participants as a challenge to collaboration. One reason for this could be due to educational silos which in turn lead to practice silos [56, 57]. However, there has been a move towards interprofessional education [56, 57] and dental public health as an approach to try and address these silos [14]. To enable collaboration in dentistry, structural changes in the field are needed [14, 53], enabling other health professionals to be involved in prevention efforts [46]. An example of how collaboration between dental professionals and public health nurses can bring about a reduction in dental caries rates in children and reduce inequalities is the ‘childsmile’ initiative in Scotland. A settings approach was used where strategies such as tooth brushing program and fluoride varnish application were undertaken in schools and nurseries [58, 59].

## Limitations

Limitations of this study include not pilot testing the questions for the NGT and the inability to probe for further detail during the NGT section of the study. To account for this, the email data collection stage was added, however, there was a low response rate. As only six of the twenty-four participants responded to the second stage of data collection, there may be some response bias. The participants who responded may have a strong opinion/passion about health promotion and therefore the results may be skewed. Furthermore, demographics of the participants were not collected during data collection. Therefore, no discussion can be had on whether the participants characteristics could have influenced the data. Another limitation was that the stage 4—generating ideas section of the data collection was not audio recorded. Key discussions may have been missed if they were not captured by the scribe.

## Strengths

Despite these limitations, this study has used a data collection method which has been underutilised in the field of dentistry. This method allowed for a wide range of ideas to be presented without participants being influenced by each other. Additionally, this study outlines the views of oral health academics in this topic, who are an under researched population group.

## Study implications

This study highlights that oral health academics understand the broad nature of health promotion, but still focus more on behavioural approaches, this may be influencing dental and oral health students understanding of health promotion and perpetuating the reliance of behaviour change approaches in dentistry. Further research into what health promotion training and the amount of time spent on upstream or downstream approaches is being provided to dental and oral health students is needed. Participants in this study emphasized the need to collaborate with other health professionals. This collaboration needs to start in university training to embed this practice, so students continue to collaborate once graduated.

## Conclusion

The findings of this study demonstrate oral health academics understand the breath of health promotion strategies, but still focus more on behavioural change approaches to health promotion, which is not uncommon within the dental field. Both individual and population health promotion approaches need to be utilised in dentistry and oral health to prevent dental disease. For a change to occur the structure and curative culture of dentistry needs to be challenged. Funding models for dentistry need to incorporate health promotion interventions and not just focus on restorative treatment. Competencies set out by accrediting bodies for dental and oral health education need to reflect the breadth of health promotion and training of dental professionals needs to become more holistic and move beyond behaviour change and individual prevention strategies. Opportunities for collaborating with other health professionals using the common risk factor approach need to be embraced and breaking down of the silos of practice needs to be addressed. These improvements and changes need to start at the education of future dental professionals.

## Data Availability

The datasets used and/or analysed during the current study are available from the corresponding author on reasonable request.

## Abbreviations

NGT: Nominal Group Technique
ICOHIRP: International Centre for Oral Health Inequalities Research and Policy
COHA: College of Oral Health Academics
